# There Are No Insurmountable Barriers: Passage of the *Helicobacter pylori* VacA Toxin from Bacterial Cytoplasm to Eukaryotic Cell Organelle

**DOI:** 10.3390/membranes14010011

**Published:** 2023-12-28

**Authors:** Miroslaw Jarzab, Joanna Skorko-Glonek

**Affiliations:** Department of General and Medical Biochemistry, Faculty of Biology, University of Gdansk, Wita Stwosza 59, 80-308 Gdansk, Poland; miroslaw.jarzab@ug.edu.pl

**Keywords:** VacA, *Helicobacter pylori*, autotransporter, membrane insertion, pore-forming toxin, anion-selective pore, endosome, vacuolation

## Abstract

The Gram-negative bacterium *Helicobacter pylori* is a very successful pathogen, one of the most commonly identified causes of bacterial infections in humans worldwide. *H. pylori* produces several virulence factors that contribute to its persistence in the hostile host habitat and to its pathogenicity. The most extensively studied are cytotoxin-associated gene A (CagA) and vacuolating cytotoxin A (VacA). VacA is present in almost all *H. pylori* strains. As a secreted multifunctional toxin, it assists bacterial colonization, survival, and proliferation during long-lasting infections. To exert its effect on gastric epithelium and other cell types, VacA undergoes several modifications and crosses multiple membrane barriers. Once inside the gastric epithelial cell, VacA disrupts many cellular-signaling pathways and processes, leading mainly to changes in the efflux of various ions, the depolarization of membrane potential, and perturbations in endocytic trafficking and mitochondrial function. The most notable effect of VacA is the formation of vacuole-like structures, which may lead to apoptosis. This review focuses on the processes involved in VacA secretion, processing, and entry into host cells, with a particular emphasis on the interaction of the mature toxin with host membranes and the formation of transmembrane pores.

## 1. Introduction

*H. pylori* is a prevalent Gram-negative, spiral-shaped human gastric pathogen whose estimated carrier rate is 50% worldwide. The *H. pylori* infections are usually asymptomatic. However, this bacterium is also known as a major contributing factor leading to chronic inflammation of the gastric mucosa, development of gastric ulcers, and cancer [1]. *H. pylori* produces several virulence factors of which VacA (vacuolating cytotoxin A) and CagA (cytotoxin-associated gene A) are most intensively studied.

The most plausible functions of VacA are to enhance the availability of nutrients (or other factors, such as metals) and interference with the functions of the immune system. Those actions of VacA assist bacterial colonization, survival, and proliferation during long-lasting infections [2]. VacA exerts various effects on mammalian cells by affecting functions and the integrity of the plasma membrane and membranes of other organelles [3]. The most notable effect of the VacA protein on the host cells is the formation of large cytoplasmic vacuole-like structures [4,5]. The mechanism of vacuole formation by VacA is dependent on its ability to form channels across membranes. As such, VacA represents the largest class of bacterial toxins, i.e., the class of pore-forming toxins (PFTs) [6]. The target of a PFT is the plasma membrane, also known as the cell membrane, whose role is to maintain cell integrity and separate the cell contents from the external environment while ensuring the exchange of chemical compounds, energy, and information [7,8]. A disruption of the selective permeability of the cell membrane, regardless of the cause, usually leads to the loss of valuable substances from the cell. However, it may also allow the entry of harmful substances, significantly affecting cellular homeostasis and the ability to detect and respond to external stimuli [9]. After internalization by the host cell, VacA creates ion channels in endosomal membranes and leads to the formation of vacuoles. Moreover, it penetrates the membranes of mitochondria and other organelles, causing changes in their functioning.

**Figure 1:**
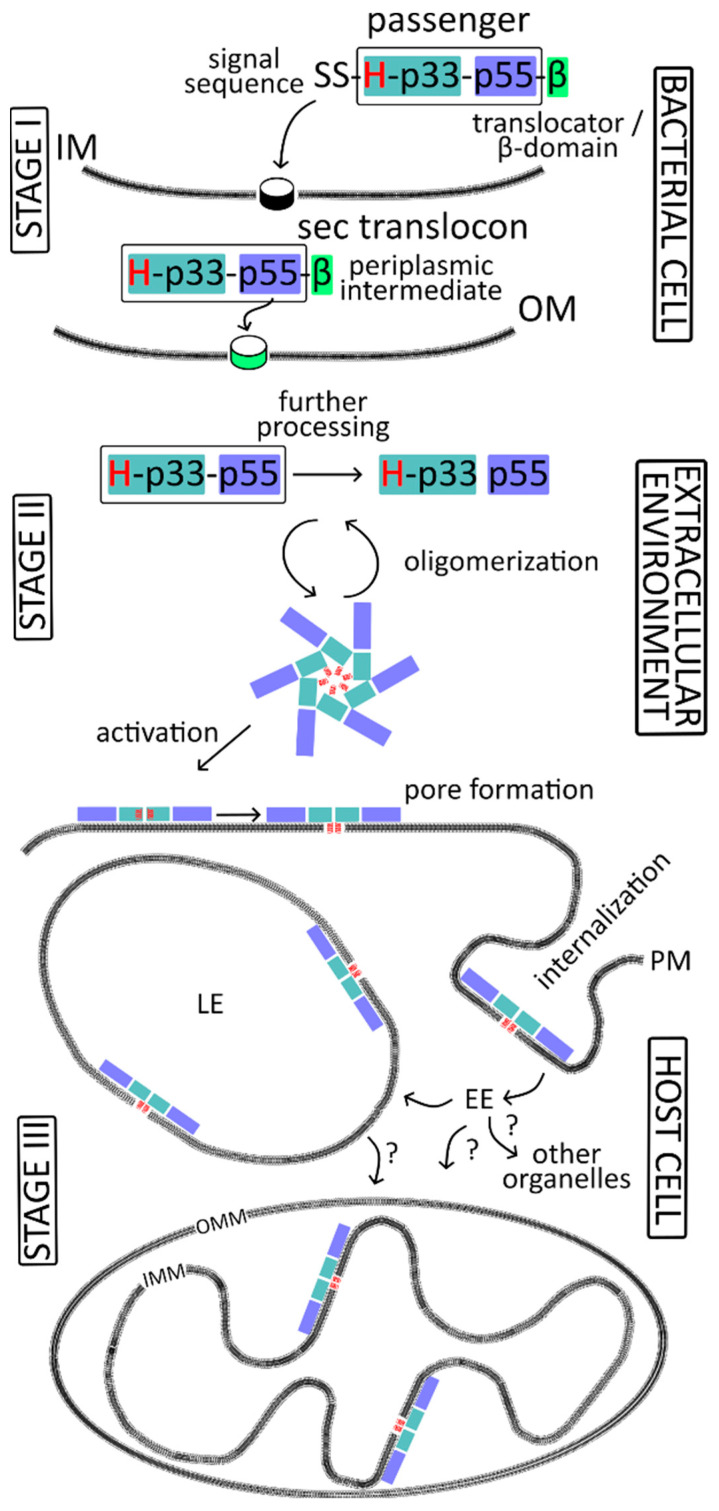
An overview of the fate of VacA in the bacterial and host cells. Stage I. The precursor of VacA is synthesized in the cytoplasm of the *H. pylori* cell, and it contains a signal sequence (SS) for the SEC-dependent translocation across the inner membrane (IM), the passenger domain with two subdomains of the toxin (p33 and p55), and the translocator β-domain (shown in green). The passenger domain crosses the outer membrane (OM) via a pore in the translocator β-domain. Stage II. The released passenger domain is further processed to form a mature toxin that undergoes a reversible oligomerization process. Stage III. The toxin interacts with the host cell plasma membrane (PM). The N-terminal part of the p33 domain (marked as red H) is incorporated into the lipid bilayer and participates in the formation of the pore. VacA becomes internalized in the process of endocytosis, and it can be found in late endosomes (LE), inner mitochondrial membranes (IMM), and the membranes of other organelles. Question marks denote the hypothetical mechanisms that still lack unequivocal experimental evidence. Based on [10,11,12,13].

VacA is an excellent example illustrating the ability of a protein toxin to overcome various membrane barriers in order to reach its target location and induce appropriate effects. However, before the active toxin causes vacuolation in host cells, it must undergo a “journey,” during which it is subject to many post-translational modifications and conformational changes. The protein is synthesized as a precursor containing a signal peptide at the N-terminus, directing it to the periplasm, and a translocator domain at the C-terminus, which is responsible for the secretion of the toxin out of the cell. Between these regions is the passenger domain that contains the actual toxin. The toxin is cut out from the precursor sequentially while crossing the individual membranes of the *H. pylori* cell, which include the inner membrane (IM) and the outer membrane (OM). First, during transport across the IM, the signal peptide is removed. Second, another cut occurs, which frees the passenger domain. This process occurs after the passenger domain passes through the OM and causes the release of the active toxin to the extracellular environment. Finally, the toxin is cleaved into two subunits, designated p33 and p55. It should be noted that the precursor and the cleaved toxin do not have a toxic effect on the bacterial cell, only on the host cells. This means that VacA recognizes target cells by binding to the appropriate receptor on their surface. The following chapters present a detailed description of the individual stages related to the maturation, secretion, and interaction of the toxin with host cell membranes. An overview of the fate of VacA in the bacterial cell and host cell, along with the division into stages, is presented in Figure 1.

## 2. Stage I—Events in the *Helicobacter pylori* Cell

### 2.1. The vacA Gene

The *vacA* gene is found in almost all isolated *H. pylori* strains [3,10,14,15,16,17]. In the case of the *H. pylori* 60190 strain, the transcription start site of *vacA* is located 119 bp upstream of the ATG translation start codon. The transcription of this gene depends on the extended −10 motif: tgaTAAAAG, compared to *E. coli* consensus sequences: TATAAT or TTGACA, and −35 motif sequence TTTATG compared to *E. coli* consensus sequence TTGACA [18]. The mRNA stem–loop-forming structure in the 5′ untranslated region (UTR) of the *vacA* transcript was also identified, and its disruption affects the half life and level of *vacA* mRNA and the VacA protein level [19]. The expression of *vacA* was reported to be influenced by environmental conditions to which the bacterium is exposed, such as acidic pH [20], iron limitation [21], contact with gastric epithelial cells [22], or NaCl [19,23,24]. Higher VacA levels were detected in the supernatants of high-salt-treated bacterial cultures, compared to those grown in conventional media [24], and at least a part of this increase could be attributed to increased *vacA* transcription [19,23,24].

**Figure 2:**
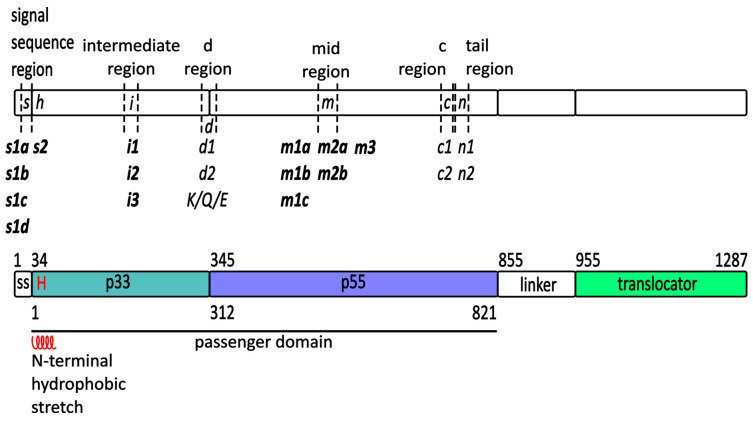
Allelic diversity in regions of the vacA gene and the VacA preprotoxin domain structure (based on VacA from *H. pylori* 60190 strain). Based on [36,37,38].

The *vacA* gene varies in length (3.9 kb ± 35 bp) among strains, is highly polymorphic, and has at least six known heterogenic regions that may influence toxin activity and contribute to variable disease outcomes. These include variability in the signal sequence (s1a-d/s2) [16,25,26], as well as in intermediate (i1-3) [27,28] and middle (m1a-c/m2a-b/m3) regions [16,25,29,30,31,32]. Three new regions were also described: d1 and d2 subtypes [33] further classified to K-, Q-, or E-types [34], a tail region, which includes n1 and n2 [34,35], and c region with c1 and c2 subtypes [35] (Figure 2).

Due to the polymorphism, only about 50% of the *H. pylori* isolates can produce detectable amounts of this cytotoxin and exhibit vacuolating activity [4,10,39,40,41].

For clarity, the description of the structure and function of VacA in this work concerns the s1m1 variant, unless otherwise stated.

### 2.2. VacA Protein in the H. pylori Cell

The *H. pylori vacA* gene encodes a precursor protein (preprotoxin) of approximately 140 kDa [10,14,15], which is known to be secreted out of the cell and released as a mature toxin.

As all proteins are synthesized in the cytoplasm, the proteins destined for secretion must cross the cell envelope, which in Gram-negative bacteria is composed of the IM, the OM, and the periplasmic space with a peptidoglycan layer between them. To ensure efficient export, several secretion pathways with specialized protein machineries have evolved (Types I–IX secretion systems). These systems facilitate translocation across both membranes and, eventually, navigation through the periplasm. Two main strategies can be used for secretion. Proteins can be secreted directly (one step) from the cytoplasm bypassing the periplasm (Types I, III, IV, VI, and VII secretion systems), or via a two-step strategy. In the latter case, the secretory protein must first pass through the IM using the general secretory (SEC) pathway or twin arginine (TAT) translocation pathway. Then, it traverses the periplasm and becomes incorporated into the OM or exported outside the cell (Type II, V, VIII, or IX secretion systems) [42]. VacA is expected to use the Type V secretion system (T5SS). Although the exact mechanism of this process is not known, the detection of VacA in the periplasm supports a two-step secretion pathway [43].

#### 2.2.1. Translocation of VacA across the Inner Membrane

**Figure 3:**
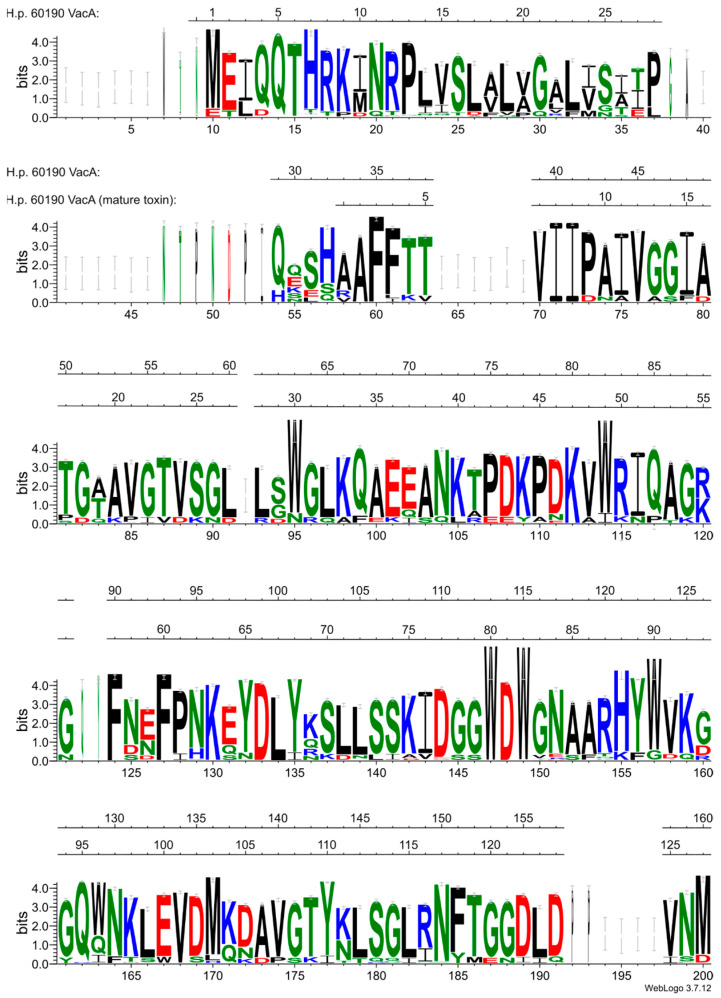
Sequence logo of signal sequence and hydrophobic motif of VacA. First 160 columns from the alignment of 3846 VacA sequences retrieved from UNIPROT using “vacA helicobacter” query subjected to Clustal X2 alignment. Logo generated using WebLogo 3.7.12 (https://weblogo.threeplusone.com, accessed on 14 September 2023). The amino acid residues are grouped and colored based on the R group of their side chain. Red denotes polar acidic amino acid residues (D, E); Blue denotes polar basic amino acid residues (K, R, H); Green denotes polar uncharged amino acid residues (C, G, N, Q, S, T, Y); Black denotes non-polar hydrophobic amino acid residues (A, F, I, L, M, P, V, W). Reference numbering for *H. pylori* 60190 VacA full sequence and mature toxin included above the sequence logo.

The recognition and binding of the signal sequence (SS) is the first and necessary step leading to protein translocation through the IM. An analysis of the VacA precursor sequence revealed the presence of a 33 amino-terminal SS with typical features recognized by the general SEC translocon including positive N-terminal charges, a central hydrophobic stretch [10], and the signal peptidase cleavage site between Ala^33^–Ala^34^ [10,15]. The amino-terminal amino acid of VacA purified from the *H. pylori* 60190 culture medium was determined as Ala^34^ [44] (Figure 3).

In *H. pylori*, the proteins responsible for the SS recognition, binding, and delivery to the SEC complex (core complex: SecYEG, SecY–HP1300, SecE–HP1203a, and SecG–HP1255 with ancillary subcomplex SecDF–YajC, SecD–HP1550, SecF–HP1549, YajC–HP1551, and YidC–HP1450) [45] have not been studied in detail. By analogy to the mechanism described for *E. coli*, it can be expected that the trigger factor homolog (HP0795) binds and protects the nascent proteins that exit the ribosome [46]. The homolog of the *E. coli* SecB chaperone, which delivers preproteins to SecA in the post-translational pathway, was not identified in *H. pylori*. An alternate, co-translational means of transport involving a signal recognition particle (SRP) homolog HP1152 and FtsY (HP0763) [47] is not likely, similar to other autotransporter proteins, but it cannot be excluded [48]. After translocation, the signal peptide will most likely be cut off by the protein HP0576 (a homolog of *E. coli* Type I signal peptidase). However, this process has not been studied in *H. pylori*.

#### 2.2.2. Periplasmic Transit

Proteins pass across the IM via the SEC system in unfolded forms [49]. Therefore, once a polypeptide reaches the periplasm, components of the extracytoplasmic protein quality control system, including a set of periplasmic chaperones and folding helpers, stabilize nascent proteins to prevent misfolding or aggregation [50]. The periplasm of *H. pylori* contains several putative chaperones showing varying degrees of homology to the *E. coli* counterparts. One example is PPIase (peptidyl–prolyl cis–trans isomerase) HP0175, which resembles the *E. coli* SurA protein [47]. SurA plays a major role in (1) The binding of the proteins exiting the Sec channel [51]; (2) Trafficking them across the periplasm; and (3) Incorporating of the β-barrel outer membrane proteins into the OM [52]. The other putative chaperones/folding factors include HP0977 (a homolog of PpiC) and a SurA-like protein HP0659. However, it should be stressed that there are no experimental data on the involvement of these proteins in the transport and folding of exported proteins in *H. pylori*, and their function has only been proposed based on their similarity to their counterparts in other bacterial species. Nevertheless, autotransporters have been shown to interact with chaperones during the periplasmic transit (reviewed in [53]). Therefore, it can be assumed that VacA is also protected in this way before it is incorporated into the OM and exported outside the cell.

#### 2.2.3. Translocator Domain and Translocation across the Outer Membrane

Based on the *vacA* gene structure, VacA has been classified in the autotransporter (AT) family of secreted proteins [10,15], i.e., Type V secretion system (T5SS). The T5SS is regarded as the simplest and one of the most common secretion systems in Gram-negative bacteria. Based on gene organization and protein structure, six sub-classes of the T5SS are distinguished (Va-f) [54]. The VacA protein is classified as Va type, i.e., classical AT [3]. Proteins of this type are composed of the N-terminal passenger domain and the C-terminal translocator (also termed autotransporter) domain, which adopts in the OM a β-barrel structure with a central transmembrane pore. Crystal structure of the translocator domain of VacA is not known, but the type Va ATs generally consist of a 12-stranded β-barrel domain that usually functions as an anchor in the outer membrane [55]. Indeed, the C-terminal fragment of VacA has a predicted β-barrel structure [56,57,58] and is required for secretion of the 88 kDa toxin [10,15,59].

Despite the name “autotransporter,” the stage of insertion and folding of the translocator in the lipid bilayer is assisted by the β-barrel assembly machinery (BAM) or translocation and assembly module (TAM) machineries. The VacA β-barrel translocator domain becomes inserted into the OM in a process that is probably assisted by the BamA/TamA homolog protein HP0655. Once in the membrane, the translocator domain promotes passage of the passenger domain to the cell surface. There, the passenger can adopt the functional conformation.

#### 2.2.4. Release of the Passenger Domain

Following translocation through the translocator channel, the passenger domain is cut off and released as the protoxin of approximately 88 kDa. However, varied masses ranging from 87 to 95 kDa were reported [10,14,15,44,60,61,62,63,64,65]. A cleavage site between amino acids 991 and 992 was determined by collision-induced dissociation mass spectrometry for *H. pylori* 26695 VacA [61], but the toxin may be processed further yielding of proteins consisting of 821 aa [60]. At present, there is considerable controversy regarding the process of VacA cleavage. Since no protease responsible for VacA processing has been identified, an autocatalytic cut was proposed. This is how the passenger domains of some *E. coli* autotransporters, such as EspP [66] and Hbp [67], are processed. There are some indications that VacA may have autoproteolytic activity as the cytotoxic activity of VacA can be blocked by treatment of VacA with 3,4-dichloro-isocoumarin (a serine protease inhibitor). In addition, some features of the amino-terminal portion of VacA are related to serine proteases [68]. However, the *H. pylori* mutant strains with deletion within the p33 region of *vacA* (but encoding p55 and the translocator domains) expressed truncated VacA, which was C-terminally processed and secreted [69,70], indicating that an intact amino-terminal portion of VacA is not required for proteolytic processing of the protoxin. Nevertheless, no direct experimental evidence that VacA possesses proteolytic activity is available.

**Figure 4:**
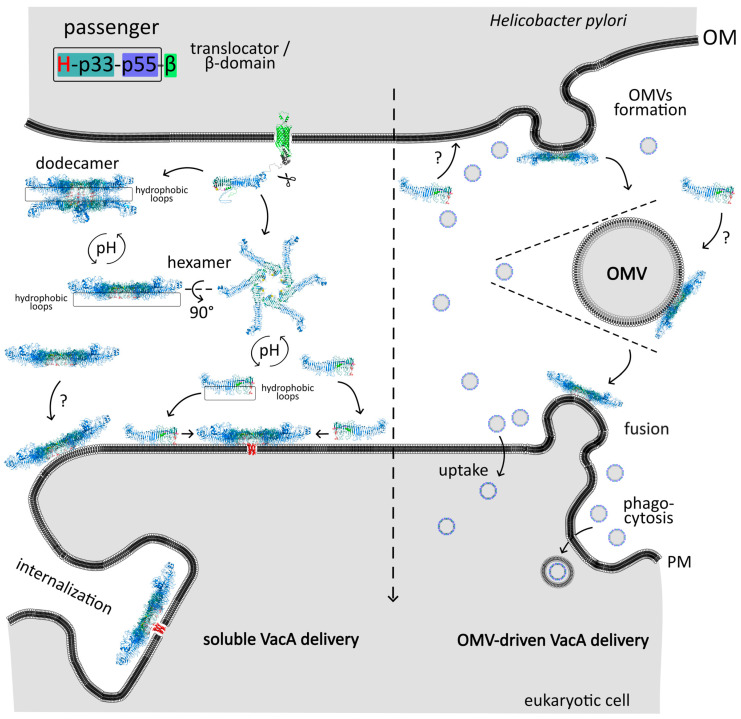
VacA in the extracellular space. The figure shows a fragment of the *H. pylori* cell (top), the extracellular space, and a fragment of the eukaryotic (host) cell (bottom). For translocation across the outer membrane (OM), the C-terminal ~33 kDa translocator domain inserts into the outer membrane and translocates the passenger domain of VacA to the external side of the outer membrane. Proteolytic cleavage results in the release of the active 88 kDa cytotoxin molecule, which can then oligomerize to form mainly dodecamers. Acidic conditions can promote disassembly of VacA oligomers, which then expose hydrophobic loops, allowing for interaction with membrane lipids. VacA can probably bind to bacterial OM or directly to OMVs to form VacA-containing OMVs (blue/gray circles), which transfer VacA to epithelial cell (by fusion, uptake, or phagocytosis of OMVs). VacA molecules bind to the surface of epithelial cells where they oligomerize, become inserted into plasma membrane, and create a pore. Membrane-bound VacA can be subsequently internalized. Pore forming α-helices are shown in red, p33 domain—deep teal, p55 -marine, passenger-translocator linker—gray, translocator—green; VacA model structure used: PDBid: 6NYG, 1SEW and AF-P55981-F1. OMVs, VacA molecules, and cellular membranes are not drawn to scale. One vesicle with bound VacA is shown in enlargement. Question marks denote the hypothetical mechanisms, which lack unequivocal experimental evidence. Based on [13,73].

It is worth noting that although the VacA protoxin is generally secreted to the extracellular environment as a soluble protein [10,15,44,60,61], some of it may remain associated with the cell surface [63,71,72] (Figure 4). Alternatively, it may become part of OMV vesicles (Figure 4, Table 1).

## 3. Stage II—Events in the Extracellular Space

### 3.1. Processing of the Passenger Domain

The 88 kDa protein can be further processed to form separate p33 and p55 fragments, which are detected in the bacteria culture supernatant [14,60,90,91]. It is now accepted that proteolytic cleavage between p33 and p55 subunits occurs primarily between amino acids A311 and K312 (mature protein numbering) of secreted VacA toxin from *H. pylori* strain 60190 (and possibly several adjacent sites) [92,93,94]. Based on the MEROPS database and an analysis of the available data on the properties of the M03.006 subclass, PepF (HP0470) was proposed as a peptidase that cleaves the linker sequence between the p33 and p55 VacA domains (within aa 311 and 320) [95]. The p33 and p55 domains copurify, suggesting that these fragments remain associated after cleavage [14]. The experiments using various techniques, including yeast two-hybrid system [94], gel filtration [96], and co-immunoprecipitation from transiently transfected cells [97], further confirmed interaction between p33 and p55 domains.

### 3.2. Structure of the Mature VacA Toxin

Although the released toxin molecule has a predicted molecular mass of ∼88 kDa, it is recovered from growth media as a large >600 kDa complex [44,96]. Using deep etch electron microscopy, native cytotoxin has been shown to form regular ~30 nm oligomers resembling hexagonal “flowers,” each composed of a ~15 nm central ring surrounded by six ~6 nm globular “petals”. The intact VacA oligomer was found to consist of 12 subunits of approximately 88 kDa that were assembled into two interlocked six-membered arrays, the overlapping of which gave a flower-like appearance [91,96]. Further analysis by cryo-negative staining of the VacA preparations showed multiple types of oligomeric VacA structures, including single-layered astral arrays, bilayered forms, and two-dimensional crystalline arrays [98]. Using cryo-negative staining electron microscopy, views of the different oligomeric structures in multiple orientations were also classified and analyzed, and three-dimensional models of the bilayered forms of VacA were constructed with a resolution of about 19 angstroms [99].

Summarizing the results obtained using various techniques, water-soluble VacA forms several oligomeric structures, such as hexamers [96,98,99,100,101,102,103,104] and heptamers [96,99,100,102,103,104]. However, they predominantly organize into double-layered oligomeric structures [102], mainly dodecamers [98,99,101,103,104] and tetradecamers [99,101,103,104].

A complete high-resolution structure of the active VacA toxin has not been obtained. Only the crystal structures of the isolated p55 subunit and the non-oligomerizing VacA variant containing the p33 subunit have been solved. Nevertheless, structural data obtained by other methods [103,104,105,106], including circular dichroism (CD) spectra of the purified VacA protein [107], confirm that the VacA toxin has predominantly a parallel β-strand structure, which is characteristic of autotransporter passenger domains.

## 4. Stage III—Events in the Host Cell

### 4.1. Interaction with the Host Cell Membranes

VacA was shown to bind to plasma membranes of different types of human/mammalian cells e.g., HeLa [90,108,109], AGS [90], G401 [110,111], AZ-521 [110,111,112,113,114], RK-13 [111], primary mouse glandular stomach epithelial cells [115], primary human T lymphocytes [116], and others. Most cellular alterations caused by VacA, like permeabilization, increased current, membrane depolarization, and ion conductivity, are attributed to membrane channel formation, either in the plasma membrane or in the membranes of endosomes, lysosomes, or mitochondria [2,101,117,118,119,120,121,122,123,124,125,126,127,128]. VacA is found in glycosylphosphatidyl inositol anchored protein (GPI-AP)-enriched early endosomal compartments (GEECs) within 10 min after internalization and within next 10ths of minutes in early endosomes (EEs), and then in late endosomes (LEs) [129]. This means that most of VacA is rapidly internalized upon contact with cells and can exert its activity on cell organelles. There is probably no release of the pore-forming moiety of the toxin into the cytosol [130]. It cannot be excluded that a fraction of VacA remains in the plasma membrane where it can also affect the cell physiology and contribute to *H. pylori* virulence.

Overall, the interaction with and internalization of VacA into target cell requires several steps: (1) Binding to the plasma membrane (via receptor); (2) Oligomerization in lipid rafts and formation of the toxin pore; and (3) Internalization by lipid raft-dependent, clathrin-independent endocytosis, or clathrin-independent carriers [130].

#### 4.1.1. VacA Receptors

The first step in pore formation by PFTs is the binding of the toxin to a receptor on the surface of the target cell membrane. The most common types of receptors for PFTs are glycan receptors, protein receptors, and lipid receptors (cholesterol in particular). The binding of the toxin via receptors increases the local concentration of the toxin and promotes oligomerization [131]. Despite years of research, the type of molecule (i.e., lipid or protein) that serves as the VacA receptor on the host cell is still not clearly defined. The interaction of secreted VacA with the target eukaryotic cell via specific surface proteins was reported for numerous cell types, e.g., epidermal growth factor receptor, EGFR (HeLa cells) [108], receptor-type protein tyrosine phosphatase α, RPTPα (kidney cells) [110], (stomach cells) [111], RPTPβ (stomach cells) [111,112,114], (gastric epithelial cells) [115], (kidney cells) [132], integrin beta chain-2, CD18 (T cells) [116], low-density lipoprotein receptor-related protein-1, LRP1 (stomach cells) [113], and Multimerin-1 (platelet cells) [133]. Non-protein molecules were also found to be bound by VacA, e.g., heparan sulphate [113,134], sphingomyelin [135,136], glycosphingolipids [137], and phospholipids [138]. Of the many putative VacA-binding receptors identified in the cell membrane, only the presence or absence of sphingomyelin affected the degree of VacA binding and cell sensitivity to the toxin [135]. Interestingly, the length of the sphingomyelin acyl chain also determines the intracellular transport of VacA [136]. Sphingolipids and cholesterol facilitate the organization of relatively small, highly dynamic, and transient plasma membrane platforms. These platforms attract specific proteins, like lipidated proteins and glycosylphosphatidylinositol-anchored proteins (GPI-APs), and are known as lipid rafts [139]. Consequently, the binding of VacA to sphingomyelin probably accounts for the localization of the toxin to lipid rafts. Several lines of evidence support this hypothesis. First, VacA has an affinity to supported lipid bilayers of various compositions ([total brain lipids], [90% sphingomyelin (SM)-10% cholesterol], [45% dioleoylphosphatidylcholine (DOPC)-45% SM-10% cholesterol], and [50% DOPC-50% SM]) [140]. Furthermore, VacA binds to lipid rafts under conditions that preclude the recruitment of new proteins, suggesting that the VacA receptor resides in lipid rafts permanently [141]. VacA was also found to be enriched in lipid rafts isolated as detergent-resistant membranes (DRMs) [114,125,129,135,142,143], and VacA localization to lipid rafts was visualized using giant plasma membrane vesicles (GPMVs) from HeLa cells [13]. Acid activation has been shown to significantly increase the efficiency of VacA binding to lipid rafts [125].

Cholesterol, an important component of lipid rafts, also influences the binding of VacA [140], as the VacA-induced cell vacuolation was inhibited by the treatment of the cells with either the cholesterol-depleting agent methyl-β-cyclodextrin [125,142,144] or the cholesterol-binding agent nystatin [125,145].

Many membrane-embedded proteins acting as receptors for exogenous ligands are either permanently residing in or are permanently excluded from lipid rafts. However, some membrane receptor proteins that normally reside outside lipid rafts can be recruited into lipid rafts upon ligand binding and crosslinking of receptor–ligand complexes [146]. VacA, after binding to its receptor RPTPβ in non-lipid microdomains of cell surface rafts, also can relocate to lipid rafts [114]. VacA–receptor complex recruitment to lipid rafts can increase toxin local concentration favoring toxin oligomerization and channel formation [145].

#### 4.1.2. Pore Formation

Pore-forming toxins can be classified as α- or β-PFTs depending on the composition of their membrane-spanning regions, i.e., out of α-helices or β-barrels [9,147]. Two alternative pore-forming mechanisms for α- and β-PFTs exist. Most α-PFTs bind to the membrane, and once a critical concentration is reached, PFT subunits insert concomitantly into the membrane and oligomerize to form the final pore. The formation of incomplete but functional pores is possible. β-PFTs concentrate at the membrane interface into the pre-pore, and once oligomerization is completed, conformational change of the toxin molecule inserts it into the membrane [131]. However, the details of the pore formation process by PFTs, including VacA, are still not completely understood [147].

##### Activation of the Toxin

When added to mammalian cells culture, water-soluble VacA exhibits little or no activity [109,112,138,148], and it needs to be activated either by acid treatment [109,112,138,148] or alkaline activation [109,112]. This phenomenon likely results from the quaternary structure of the VacA molecules. The acid or alkaline treatment results in the disassembly of VacA oligomers into monomers [91,112,138,149], influencing physical properties of VacA [148]. VacA monomers can subsequently reassemble into oligomers when in contact with membranes [102] or in solution at neutral pH [91,112,117] (Figure 4).

Changes accompanying monomer formation and reassembly into oligomers enhance interaction with and insertion into artificial membranes [117,150,151] or of target cells [112,125,142]. Therefore, it was expected that the pH treatment leads to conformational changes that facilitate membrane insertion of the toxin. However, it was demonstrated that the VacA subunit structure does not undergo gross rearrangements, and it was proposed that the dissociation of the high-order oligomeric forms is the main result of VacA acid activation [102]. The monomers can then bind cell surface via specific receptors with increased affinity compared to that of oligomerized toxin [112]. The hydrophobic regions exposed in monomers (but inaccessible in dodecamers and tetradecamers) insert into the membrane, oligomerize, and form a pore.

The ability of VacA to undergo reversible oligomerization and disassembly seems to be crucial for cytotoxic activity. The non-oligomerizing mutant variants of VacA (Δ28–108, Δ49–57, Δ56–83, Δ85–127, Δ112–196, Δ114–294, and Δ346–347) lack cytotoxic activity [70,94,106,122,123,152]. In addition, the VacA variants (K44C/E338C, K47C/E338C, K55C/D346C) locked into an oligomeric state (cysteine substitutions at sites of p33-p55 contact) are not toxic [152]. Dominant negative mutant forms of VacA (Δ6–27, Δ49–57, Δ346–347) can inhibit the activity of wild-type VacA through a process that involves the formation of mixed oligomers [70,122] or interference with the reassembly of wild-type VacA oligomers after acid treatment [123].

##### Models of VacA Interaction with Membrane and Pore Formation

**Figure 5:**
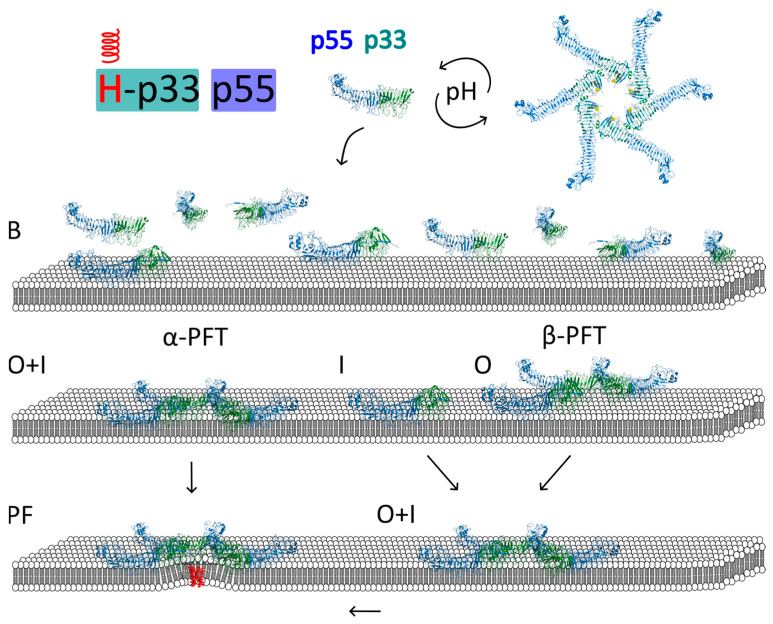
Three different models for acid-induced VacA pore formation can be distinguished based on the temporal order of toxin insertion and oligomerization: (1) Mechanisms characteristic for most of α−PFTs; in this scenario, regions of p33 insert into the lipid bilayer simultaneously with p88 oligomerization into a hexamer; (2) Regions of p33 can also insert into the lipid bilayer before p88 oligomerization into a hexamer; and (3) There is also possibility, as for β−PFTs, that regions of p33 insert into the lipid bilayer after p88 oligomerization into a hexamer and then form a pore. It should be noted that the position of the hydrophobic stretch (H) in the VacA structure is not known. Therefore, its presence was shown in the membrane-inserted oligomer only. VacA model structure used: PDBid: 6NYG and 1SEW. B, binding, I, insertion, O, oligomerization, PF, pore formation, pore forming α-helices are shown in red, p33 domain—deep teal, p55-marine. Based on [102].

Three different models for VacA functional pore formation can be distinguished based on the temporal order of toxin insertion and oligomerization (Figure 5):

1. Regions of p33 insert into the lipid bilayer simultaneously with p88 oligomerization into a hexamer
2. Regions of p33 insert into the lipid bilayer before p88 oligomerization into a hexamer
3. Regions of p33 insert into the lipid bilayer after p88 oligomerization into a hexamer

Once bound to the membrane, the p33 regions are expected to undergo structural rearrangement(s) that allow VacA to insert into the bilayer and organize into stable hexamers to form membrane pores [102]. The amino terminus of the p33 subunit contains a predicted hydrophobic region of 32 uncharged residues responsible for insertion into membrane [3,60]. This region contains several GXXXG motifs, amino acid sequences that are predicted to mediate transmembrane dimerization [126,153]. Moreover, it was demonstrated that this part of p33 is relatively unstructured in the monomeric form of VacA and becomes organized during oligomer assembly to form α-helix. This feature may facilitate insertion into the lipid bilayer and pore formation [70,126]. Substitutions of amino acids within this region, including the GXXXG motifs, abolish membrane channel-forming activity and vacuolating toxin activity [93,126,154].

**Figure 6:**
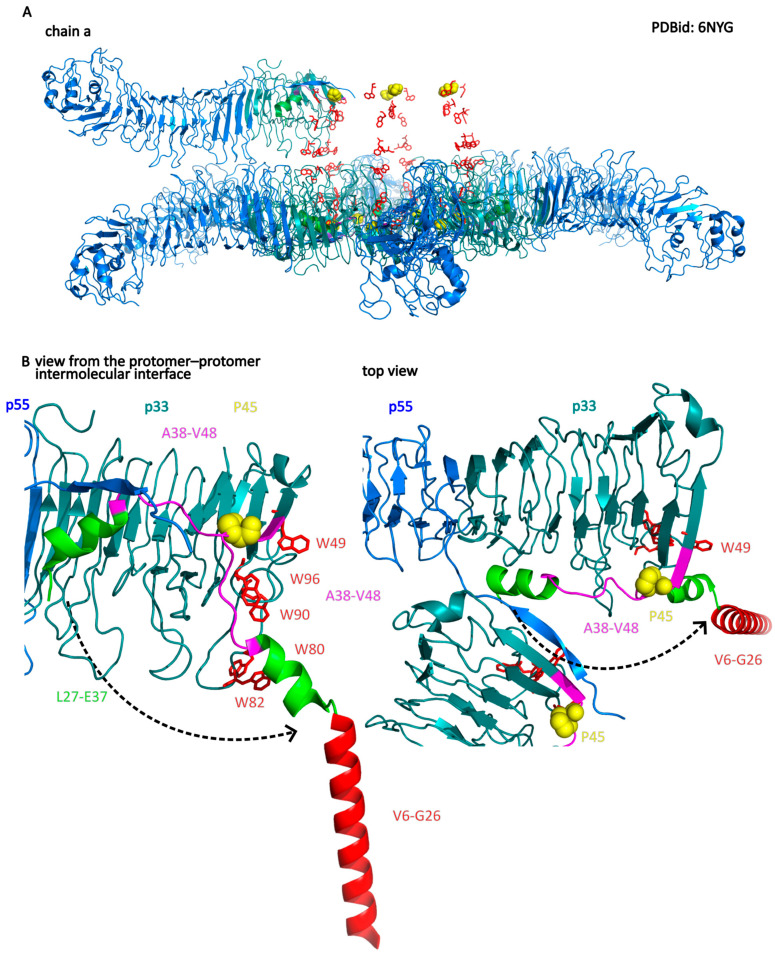
Model of the VacA dodecamer (**A**) and membrane insertion of the VacA hexamer (**B**). (**A**) Structure of water-soluble VacA dodecamer (PDBid: 6NYG). For clarity, five out of six protein chains (b–f) were removed. Structural elements were colored as follows: p33 domain—deepteal, p55-marine, L27-E37 α-helix—green, linker from helix L27-E37 to W49—magenta, tryptophan residues of the tryptophan-rich region—red, Proline 45—yellow. (**B**) After membrane binding aided by tryptophan-rich region (W49, W80, W82, W90, and W96), the N-terminal segment (helix L27–E37 and linker A38–V48 with channel-forming helix V6–G26 not visible in the structure) swings out from the protomer–protomer interface using P45 as a hinge and then exposes the hydrophobic N terminus to form the helix within the membrane (helix V6–G26 modelled in the structure in “after insertion” state). VacA model structure used: PDBid: 6NYG and 1SEW. Based on [104].

However, VacA oligomers were also shown to exhibit a weak vacuolating activity [109,112,138,148] and ability to bind to cell membranes. The membrane binding of oligomers was significantly weaker when compared to acid-activated VacA, but the protein was still targeted to lipid rafts [13]. The model of VacA interaction with membrane at neutral pH was proposed with the oligomerization occurring before insertion and pore formation [104] (Figure 6). The model assumptions are as follows:

1. Regions important for insertion into the membrane and channel formation are located at the N-terminal region of the p33 subdomain. These include helices V6–G26 and W30–E37, with the P40 residue at the loop connected to the W30–E37 helix. The W30–E37 helix is hidden in a protomer–protomer interface in the water-soluble hexamer.
2. The interaction of the VacA hexamer with the membrane initially occurs via a cluster of tryptophan residues (W49, W80, W82, W90, and W96) located near the inner rim of the hexamer and the helix W30–E37. Association with the membrane can also be mediated by electrostatic interactions between positively charged amino acid residues of the bottom side of the hexamer (facing another hexamer in dodecamer) and anionic phospholipids.
3. Interaction with membrane lipids induces a change in the position of the W30–E37 helix, while P40 acts as a hinge for this movement. This leads to the exposure of the hydrophobic N-terminus (p33 domain), which forms a helix bundle within the membrane. Importantly, the structure of most of the hexamer’s elements remains unchanged.

##### Roles of Specific VacA Regions in Membrane Binding, Oligomerization, and Membrane Insertion

The participation of individual regions of the mature toxin in processes related to membrane binding, insertion into lipid bilayer, oligomerization, pore formation, and, consequently, in virulence and induction of disease symptoms has been intensively studied by many research teams. Both subunits of the mature toxin, p33 and p55, are required for cell vacuolation and host cell binding [92,97,99,155,156]. However, each domain is responsible for different VacA effects. It is generally accepted that p33 is responsible for VacA pore formation, while p55 is believed to mediate VacA binding to receptors on host cells [155,157]. However, studies performed on various VacA toxin isoforms and mutants suggest that this functional separation is not so clear, and that both subunits, p33 and p55, participate in cell binding.

As mentioned in Section 3.1, various forms of mature toxin may arise as a result of the *vacA* gene polymorphism. There are three regions related to the degree of virulence of bacteria and the effects they cause on host cells: “s,” “i,” and “m”.

1. The “s” region (signal region), localized at the N-terminus of the VacA preprotein, is identical to the signal sequence in the s1 subtype (and is completely removed during export from the cytoplasm), while in the s2 subtype, the cleavage site of the signal sequence is different. As a result, the VacA protein is longer by 12 amino acid residues at the N-terminus. Its processing during VacA export affects the ability of membrane insertion and pore formation by the secreted toxin. Consequently, the N-terminal sequence of the mature toxin in the s1 type is different than that of the s2 type. This is associated with the different properties of both forms. The s1 type fully exhibits vacuolating activity, while the s2 type lacks detectable cytotoxic activity [158].
2. The “i” region (intermediate region) placed at the C-terminus of the p33 domain has been reported to be involved in both cell vacuolating and binding to various cell types [27,159].
3. The “m” region (middle region) located within the p55 domain with the most common types (m1 or m2) is associated with differences in the ability of VacA to bind to distinct cell types [111,160,161] and exhibit cytotoxin activity [16,111,160,161,162]. The m1 and m2 forms seem to have different cell-binding specificity. The m2 form induces vacuolization in the primary gastric cells of the RK-13 cell line, but, contrary to the m1 form, it is not able to cause vacuolization in HeLa cells [160]. Several different chimeric variants in the m1/m2 mid-region (R460-G793) of VacA were tested for vacuolating activity and confirmed differences between the m1 and m2 forms of VacA in inducing vacuole formation in RK-13 and HeLa cells [163,164,165]. The experiments allowed for the specification of amino acids 460–569 within the p55 domain to be responsible for cell binding [164] and showed no significant role of the m2 variant 21-amino acid insert on vacuolization activity of VacA [163]. Different posttranslational modifications of RPTPα from HeLa may be responsible for the reduced susceptibility to m2 VacA [111]. These data favor a protein receptor-mediated interaction of VacA with plasma membrane.

There are multiple studies showing the correlation of VacA polymorphic variants with the severity of diseases attributed to *H. pylori*. This correlation was shown for gastritis [166,167], peptic ulcer [16,166,168,169,170,171,172,173], gastric cancer [27,33,169,172,173,174,175,176,177,178,179,180], and intestinal metaplasia [167,177,179,181].

##### VacA Pore Structure and Function

The formation of a membrane pore by VacA is dependent on p33. However, both domains, p33 and p55, are necessary for efficient cell binding and insertion into the membrane [155]. The structural model of the VacA hexameric anion-selective pore was generated. According to the model, the N-terminus of each VacA hexamer subunit, VacA-transmembrane (TM) (Residues 1–32) hydrophobic stretch, is expected to traverse the membrane as an α-helix. Six of the Gly residues in three GXXXG motifs pack against small Ala or Val side chains to generate the pore [182]. The importance of GXXXG motifs (V^12^xGxxxGxxxGxxxGxL^28^) was demonstrated using deletion mutants Δ6–27 [70,94], Δ1–17, Δ14–17 [93], Δ1–23/Δ673–913 [183] within the hydrophobic stretch, resulting in the loss of vacuolating activity of VacA. The G14 [93,126,183] and G18 [126] residues were demonstrated as essential for membrane channel formation and VacA cytotoxicity. The VacA Δ6–27 mutant also fails to form membrane channels. Compared to WT VacA, it shows a lack of an organized p33 central core [101]. The importance of the proper N-terminus of the mature VacA toxin is additionally underlined by the fact that the “s2” variant with the 12 amino acid N-terminal extension shows alterations in pore formation and vacuolation activity [158]. The structural model of VacA pore proposed by (Kim et al., 2004) [182] was partially confirmed by cryo–EM structural study where it was possible to trace the N terminus of p33 starting from residue L27. The visible N-terminal region of p33 consists of an α-helix W30–E37, followed by a long loop connected to the β-helix p33 domain central core [104] (Figure 6). The formation of the α-helix by the residues V6–G26 of each VacA oligomer subunit is also expected, but there is still a lack of experimental evidence of the functional VacA pore structure.

Modelling the structure of the VacA pore and studying its properties revealed similarity to other known channels. (Kim et al., 2004) [182] used the structure of the MscS (mechanosensitive channel of small conductance) protein from *Escherichia coli* containing the A^98^xxGAxGxAxGxA^110^ motif, similar to the VacA’s hydrophobic stretch V^12^xGxAxGxAxGxV^24^, to conduct a quality test of the modeling algorithm used in VacA–TM, which allowed for the validation of the model [182].

The VacA channel exhibits electrophysiological properties of the host chloride channels (ClC). Similar magnitudes of conductance, ion selectivity, and localization within eukaryotic cells largely mimic the electrophysiological behavior of channels in the host cells with a difference only in the membrane potential at which it closes. This feature allows VacA to perturb the homeostatic ionic balance across a membrane without necessarily jeopardizing vitality [6]. Interestingly, VacA is also similar to the cystic fibrosis transmembrane conductance regulator (CFTR). Both proteins form anion-selective (Cl^−^), low-conductance pores. Moreover, the conductance of VacA channel can be in the same range as those produced by CFTR stimulation. The similar biophysical properties of VacA and CFTR, as well as the ability of both proteins to penetrate the plasma membrane of respiratory cells and their presence in the endosomal compartment, suggested the use of the VacA protein as a tool to elucidate the different roles of CFTR in the pathogenesis of the CF lung disease [184].

The VacA-induced permeabilization of cells is attributed to the formation of VacA channels in the plasma membrane [118,119,120]. Channel formation is accompanied by several phenomena such as membrane depolarization [118,125,126,127], increased current detected in the cell plasma membranes [118] or lipid bilayers [117,119,120,185], and ion conductivity. The conductivity of the VacA channel is very low, and, depending on the experimental conditions, values between 10 and 30 pS were obtained [107,118,119,120]. VacA channels can conduct chloride [118,119,120,124], bicarbonate [120,124], and small organic molecules [120], including the passive transport of urea [186]. The VacA-dependent increase of current conduction was effectively inhibited by the chloride channel blockers [118,119,121,185,187] or the chemical modification of VacA [120]. The inhibitors of anion-selective channels [118,121,141] or chemical modification [120] block VacA cytotoxicity without affecting cell binding and endocytosis. Chloride channel blockers also effectively inhibited toxin-induced urea flux [186].

The results mentioned above collectively support the dependence of VacA cytotoxicity on membrane channel formation and indicate that the amino-terminal hydrophobic region of VacA plays an essential role in both membrane channel formation and cytotoxicity.

#### 4.1.3. Internalization

**Figure 7:**
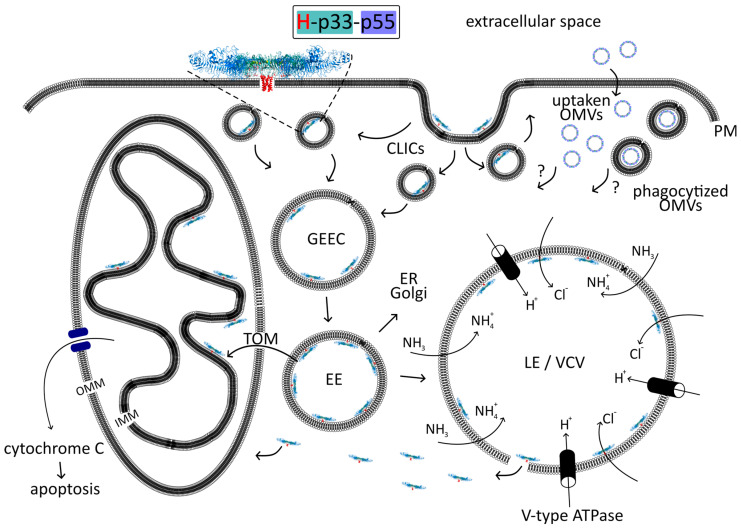
VacA intracellular trafficking. VacA binds to the cell surface and forms a channel that is subsequently endocytosed. The source of intracellular VacA can be also uptaken or phagocytized OMVs, but mechanisms of OMV VacA delivery to target sites are unknown. The first vesicles derived directly from the cell surface and exploited by VacA are termed primary uncoated clathrin-independent tubulovesicular carriers (CLICs). Endocytosis is dependent on Cdc42 and independent of clathrin. VacA accumulates in an early endosomal compartment enriched in GPI-anchored proteins (GEEC). Those compartments are formed by fusion of CLICs. GEECs mature into early endosomes (EE), and then to late endosomes (LE). VacA in cooperation with V-type ATPases causes swelling of the endosomes and, thereby, a formation of vacuoles. VacA can be released from the internal vesicular compartment by rupture of vacuoles and reach a further target, e.g., mitochondria. Another way to reach mitochondria may be the transfer of VacA from EEs during their attachment to the mitochondrial surface. VacA eventually accumulates in the mitochondrial inner membrane. Dissipation of the mitochondrial membrane potential by VacA forming chloride channels causes recruitment of Bax and Bak, release of cytochrome c, and apoptosis. Pore-forming α-helices are shown in red, p33 domain—deepteal, p55 domain—marine. VacA model structure used: PDBid: 6NYG & 1SEW. The fate of internalized or phagocytized OMVs is the subject of ongoing research and, as such, is marked by question marks. Based on [12,196].

Once bound and inserted into the plasma membrane, VacA becomes efficiently internalized [90,109,141,188,189]. VacA can be released to the extracellular environment not only as a soluble protein but also as a component of the *H. pylori* outer membrane vesicles (OMVs), including particles 20–500 nm in size that are derived from the OM of the Gram-negative bacteria [190] (Figure 7). VacA was detected numerous times in OMVs from different *H. pylori* strains (Table 1; [73,88]), vesicles from bacterial culture and infected cell lines, vesicles from patients infected with *H. pylori* [88], and in gastric juice samples of infected individuals [81]. It was established that approximately 25% of the produced VacA are packaged into OMVs [191]. It has been shown that *H. pylori* vesicles attach to, and are rapidly consumed by, epithelial cells [43,74,78,192,193]. VacA can also be delivered to cells using OMVs as a carrier [74,81,192,193,194,195], and the presence of VacA in the OMVs increases rate of their internalization [193]. The OMV’s VacA was shown to be biologically active as vacuolation was observed after incubation of cells with OMVs containing VacA [75]. However, OMV’s VacA is less effective in this respect compared to the free soluble form [191].

VacA delivery to cells from OMVs was confirmed by several experiments. Orally administered OMVs can enter gastric epithelial cells in the stomach of mice [81], as well as attach to and internalize into primary human antrum cells [192] or human gastric tubular adenocarcinoma [74]. The process of *H. pylori* OMVs internalization is relatively fast as all associated OMVs localize to cells within 20 min. Interestingly, the presence of VacA in the OMVs stimulates their uptake [193]. The exact mechanism of OMV internalization is not known. OMVs can be internalized via clathrin-dependent [193] and clathrin-independent mechanisms [193,194]. Cholesterol is hypothesized to be involved in the internalization of *H. pylori* OMVs by AGS cells, as OMV uptake was significantly reduced due to the depletion of cholesterol in the cell membrane or disruption of cholesterol-rich lipid rafts [194,195].

Soluble VacA internalization depends on the presence of lipid rafts in the plasma membrane [114,125,135,142,143,144]. Raft-dependent endocytosis encompasses various pathways but can be generally defined as the cholesterol-sensitive, clathrin-independent (CI) internalization of ligands and receptors from the plasma membrane [197]. It was demonstrated that VacA is composed of host epithelial cells via the clathrin-independent mechanism [129,145,198], while both cholesterol [140] and sphingomyelin [129,135,145,198] are important for binding to cell membranes. Other factors were also recognized as necessary for the uptake of VacA, i.e., actin, calpain, or various PKC [11]. The involvement of actin in the internalization appears to be a common feature of all clathrin-independent endocytosis pathways. VacA was not uniformly associated with the cell surface but was mostly found at the cell’s leading edges on filamentous actin (F-actin)–rich membrane extensions whose formation was controlled by the small GTPase Rac1 [129]. Rac1 regulates the reorganization of the actin cytoskeleton and intracellular signal transduction and was shown to regulate VacA activity [129,198,199]. Actin-dependent internalization of the toxin is required for vacuolization because the disruption of the actin cytoskeleton retains VacA on the cell surface [129] or inhibits vacuole formation [129,141,145,200]. VacA internalization does not differ significantly between epithelial cells and lymphocytes and involves clathrin-independent endocytosis, Rac1, and Cdc42 [198].

### 4.2. Spread of VacA in the Host Cell

A detailed analysis of VacA internalization revealed that the CLIC/GEEC endocytic pathway is involved in this process [129,201] (Figure 7). The CLIC/GEEC pathway was discovered relatively recently. It is clathrin-independent, dynamin-independent, actin-dependent, and it involves Cdc42 to form CLICs, i.e., primary uncoated clathrin-independent tubulovesicular carriers [202]. CLICs are the first vesicles derived directly from the cell surface and accommodate lipid-anchored proteins such as GPI–APs (glycosylphosphatidylinositol–anchored proteins) and a major fraction of the internalized fluid phase. Once formed, CLICs fuse to form a specialized early endosomal compartment called the GPI–AP enriched endosomal compartments (GEECs) [202].

#### 4.2.1. Endosomes

##### CLIC/GEEC (GPI-AP) Endocytic Pathway of VacA

The VacA exploits the CLIC/GEEC endocytic pathway to reach endosomes and its site of action (Figure 7). VacA was shown to accumulate in GEECs [129,136,201]. A lipid-based sorting mechanism has been proposed for GEECs as cholesterol and sphingolipid levels affect endocytosis via this pathway [202]. Cholesterol and sphingomyelin are important not only for VacA binding to cell membranes [135,140], but they also are suggested to participate in the regulation of the trafficking of VacA within cells [136,144]. For example, normal cells containing a high proportion of long acyl chain sphingomyelin (C18) direct VacA to the GEEC pathway, while cells artificially enriched in short-acyl-chain sphingomyelin (C2) recycle VacA back to the plasma membrane in a Cdc42-independent fashion [136]. Additionally, VacA-induced cell vacuolation is inhibited by the treatment of the cells with either the cholesterol-depleting or cholesterol-binding agents [125,142,144,145].

Endosome maturation is accompanied not only with changes in phosphatidylinositol phospholipids but with the differential recruitment and activation of Rab family GTPases, which control membrane identity, function, and trafficking [203].

##### Early Endosomes

Most GPI-anchored proteins are recycled back to the plasma membrane [204]. VacA avoids this step, and within 30 min, the toxin reaches early endosomes (EEs) [129] characterized by coating with the small GTPase Rab5. Once VacA reaches EE, VacA-containing vesicles become bridged to filamentous actin (F-actin) structures with the participation of CD2-associated protein (CD2AP). This allows them to exhibit high mobility and be distributed throughout the cell until they finally reach late endosomes (LE) [201]. There are also results suggesting that VacA can be directed from EE to mitochondria by the mechanism that relies on F-actin-driven vesicular motility mechanism [205]. The inhibition of F-actin by cytochalasin D blocks VacA-induced alteration in mitochondrial morphology and significantly decreases apoptosis [200], further supporting the reliance of VacA delivery to mitochondria on F-actin.

##### Late Endosomes/Lysosomes

Within 120 min, VacA is found in late endosomes (LEs) [129] where it forms anion-selective channels [117,118,119,120,121] and exhibits its hallmark feature i.e., the ability to induce the development of large membrane-bound vacuoles [4,5], termed VacA-containing vacuoles (VCVs) [11]. The membranes of the VacA-induced vacuoles contain small GTPase Rab7, a marker typically found in membranes of late endosomes (LEs) [11,206,207,208,209,210,211], which suggests that the vacuoles arise from late endosomal compartments [212]. The vacuoles also contain lysosomal (LY) markers, i.e., LAMP1 (Lgp120) [209,211] and LAMP2 (Lgp110) [208], but they do not contain markers for early endocytic compartments [206,207,209]. Therefore, the VacA vacuole is hypothesized to be a hybrid endolysosomal compartment [11]. Another protein colocalizing with Rab7 on vacuolar membranes is vacuolar H^+^ ATPase (vacuolar-type ATPase, V-ATPase), which is essential for the VacA-driven vacuole formation [207,213,214,215,216]. During endosome maturation, the lumens become increasingly acidic due to the activity of the membrane-embedded V-ATPase. V-ATPases pump hydrogen ions into the vacuole lumen, leading to a pH decrease [203]. The activity of V-ATPase is stimulated by an increase in the intraluminal chloride concentration [216,217], which is dependent on the capacity of VacA to form channels in LE [70,93,118,121,126,216]. The transmembrane pH gradient is needed for the formation and growth of vacuoles [207,213]. A strongly acidic environment leads to the protonation of membrane-permeant weak bases that diffuse into the LEs where they become trapped. This results in water influx, an increase of the osmotic pressure in these compartments, and cell swelling [39,120,188,217,218]. The presence of the weak base NH_4_Cl in the culture medium significantly increases toxin vacuolation activity [188,217,219,220]. Vacuoles virtually identical to those observed in VacA-treated cultured cells also occur in superficial gastric epithelial cells biopsied from *H. pylori*-infected patients [221,222,223] and can be observed in mice infused with VacA from surgically implanted intragastric catheters in parietal cells within gastric tissue [224].

VacA induces considerable vacuole growth until most of the cell cytoplasm is occupied by a few large vacuoles. This growth can be only sustained by the continuous addition of the membrane. One possibility is the use of endosome and lysosome interaction pathways, leading to direct fusion [225]. This scenario is supported by the recognition of several factors known to regulate fusion events between vesicles in the late endocytic pathway, e.g., Rab7, which is required for vacuole formation [209,211,226]. In addition, functional interactions between Rab7 and its downstream effector, Rab-interacting lysosomal protein (RILP), are also necessary for the formation of these bacterial compartments [211]. Another group of proteins involved in fusion processes are SNARE proteins (soluble N-ethylmaleimide-sensitive factor (NSF) attachment protein receptor). One of them, syntaxin-7, which is localized on both late endosome and lysosome, plays a crucial role in their heterotypic fusion and was suggested to be involved in the intracellular vacuolation induced by VacA [227]. Another SNARE protein, VAMP7, is localized to the VacA-induced vacuoles. The expression level of VAMP7 was enhanced in VacA-intoxicated cells, and the downregulation of VAMP7 resulted in the inhibition of VacA-induced vacuolation. VAMP7 was identified as a partner of syntaxin-7 and VacA [228]. The role of SNARE proteins in VacA-induced vacuolization should be further investigated, as another study excluded the involvement of α-SNAP and syntaxin-7 in this process. [212]. Another source of membrane required for the gradual enlargement of vacuoles may be the inner membrane of the organelle, which would fuse with the LE/nascent vacuole membrane [212,216]. This view is supported by the observation that vacuoles are largely devoid of intravesicular membrane structures, characterizing the late endosomal and lysosomal compartments from which vacuoles originate [212,229,230]. They also lack lyso–bisphosphatidic acid, a key component of such internal membrane structures [212].

The physiological role of VacA-induced vacuolation in the pathogenesis of *H. pylori* infection is still unclear. Nevertheless, vacuolation affects a number of cellular functions and may, therefore, be beneficial to bacteria. First of all, VacA leads to the partial neutralization of the acidic pH of the lumen of endosomes and lysosomes. The internal pH of vacuoles was estimated to be at least 0.4 pH units higher than that of LEs and lysosomes in the toxin free cells [231]. This change may result in the inhibition of the degradative power of late endosomal/prelysosomal cargo (e.g., EGF) and mistargeting of acidic hydrolases, e.g., procathepsin D. [231]. VacA itself is also not degraded in the lysosomes [74]. *H. pylori* is a facultative intracellular pathogen and can invade gastric epithelial cells. Intracellular *H. pylori* has been reported within VacA-dependent large vacuoles in a manner associated with an increased survival of bacteria in the cells. Hence, the vacuoles may provide an intracellular niche [232,233,234]. The suitability of these vacuoles as an intracellular niche for *H. pylori* may be due, at least in part, to the occurrence of VacA-dependent changes in the sorting of lysosomal hydrolases during vacuole biogenesis. This creates nondegradable compartments, preferentially improving the long-term intracellular survival of *H. pylori* [208,211,231]. Additionally, VacA impairs an endolysosomal calcium channel TRPML1 (transient receptor potential membrane channel mucolipin 1) activity, leading to the inhibition of lysosome and autophagy killing, which promotes bacterial colonization [233]. TRPML1 activation in the *H. pylori*-infected gastric cells or human gastric organoids reverses the toxic effects of VacA and leads to the efficient killing of intracellular *H. pylori* [233,235]. Vacuolation has also been suggested to be a simple mechanism for the release of VacA from the internal vesicular compartment. In this way, the toxin can enter the cytosol and reach a further target, e.g., mitochondria [236]. Finally, vacuolation can be considered just as a side effect of the toxin activity routed through affected compartments, as in the case of classical A–B toxins [188]. VacA exhibits a pore-forming activity, and this results in the enlargement of resulting hybrid endosomes and lysosomes before it reaches its destination [130].

#### 4.2.2. Mitochondria/Apoptosis

The presence of *H. pylori* in the gastric mucosa causes cell apoptosis [237], and VacA is one of the factors that induces this process [113,200,238,239,240,241,242,243,244,245]. A significant proportion of VacA can be detected in mitochondria [107,189,200,210,238,246], suggesting VacA might act directly on these organelles to release proapoptotic signals. The details of the pathway for targeting VacA to mitochondria are not fully understood. It was proposed that VacA can reach mitochondria after vacuole rupture and release into the cytosol, or it can be transferred to the mitochondrial surface during direct contact of endosomes with this organelle. Both pathways should eventually lead VacA to reach the mitochondrial inner membrane, where it can form anion-selective channels. VacA can also act on mitochondria indirectly by influencing the activity of the Bcl-2 family proteins, e.g., Bcl-2 (B-cell lymphoma 2) [210,238,243,247,248], Bcl-XL (B-cell lymphoma-extra large) [247,248], Bax (Bcl-2-associated X protein) [210,241,243,246,249], or Bak (Bcl-2 homologous antagonist/killer) [210,246]. Bax and Bak may also play important, direct roles in facilitating the transfer of VacA from endosomes to mitochondria. It is known that VacA leads to the activation of Bax and Bak pro-apoptotic factors [210,246]. The mechanism by which VacA induces Bax or Bak activation is not fully understood. However, it was found that VacA channel formation is necessary for this activity. The mutant variants in the channel-forming region (P9A or G14A) do not induce the accumulation of Bax on endosomes. VacA and Bax colocalization leads to an exposure of the Bax C-terminal and hydrophobic membrane-penetrating α-helice, which then becomes inserted into the outer mitochondrial membrane (OMM). The presence of Bax on endosomes, together with the physical proximity of VacA-containing vesicles and mitochondria, allows for the endosome–mitochondria juxtaposition and transfer of VacA from endosomes to mitochondria. Bax- and Bak-deficient cells were unable to cause the endosome–mitochondria juxtaposition, and the VacA was not retrieved in mitochondria [246].

For proteins to cross the outer mitochondrial membrane, they must pass through the Translocase of the Outer Mitochondrial membrane (TOM complex) [250]. The TOM complex forms a channel that has a diameter insufficient for the translocation of folded proteins, and VacA must be unfolded before or during translocation [128]. Transferring to the mitochondria is possible only if VacA contains the intact N-terminus of 32 residues [107,246], which probably acts as a mitochondrial-targeting signal for interactions with the TOM complex. VacA (at least p33 domain) is eventually integrated into the mitochondrial inner membrane (IMM) [107]. Those findings are supported by the fact that the expression of the p33 domain in cells triggers apoptosis [107,238,251]. However, (Foo et al., 2010) [252] showed that the deletion of residues 6–27 from the p33 domain did not hinder the import of the p33 subunit into mitochondria but was important for the stable integration of the subunits into the inner mitochondrial membrane [252]. There is some indication that the subunit p33 is usually accompanied by p55 [238,246,252], although the p55 subunit can be imported into mitochondria alone via internal targeting signals. However, p55 remains stably associated with mitochondrial membranes only when p33 is present [252].

VacA induces multiple negative effects on mitochondria, including mitochondrial fragmentation [251], reduction [189,210,253,254], or the irreversible loss of mitochondrial transmembrane potential [12], as well as the depletion of ATP [253]. Collectively, it leads to serious disturbances in cell metabolism. The VacA effects on mitochondria are most probably due to the presence of VacA channels in mitochondrial membranes as the VacA variants unable to form channels (Δ6–27, chimeric s2/m1 toxin [240], and VacA P9A [246]), did not induce apoptosis. Additionally, VacA mutants P9A, G14A, Δ6–27 [189,255], and S2M2 variant [189] were also defective in mediating cytochrome c release [189,255] and did not reduce the mitochondrial transmembrane potential [189]. Similarly, it was also shown that chloride channel blockers inhibit the VacA-induced cytochrome c release [189,255] and reduction of mitochondrial transmembrane potential [189]. The disruption of the mitochondrial transmembrane potential occurs prior to mitochondrial outer membrane permeabilization (MOMP) [189]. The dissipation of the mitochondrial transmembrane electrical potential results in mitochondrial recruitment and activation of dynamin-related protein 1 (Drp1), which is a crucial regulator of mitochondrial fission through its GTPase activity and causes mitochondrial fragmentation (with a disappearance of the typical spaghetti-like morphology). In cells infected with an *H. pylori vacA* knockout strain, no mitochondrial fragmentation was visible. The inhibition of Drp1-dependent mitochondrial fission within VacA-intoxicated cells prevents the activation of Bax, MOMP, and, consequently, cell death [251]. As mentioned earlier, VacA can also act on mitochondria indirectly by influencing the activity of the Bcl-2 family proteins. VacA was shown to upregulate the expression of Bax (a multi-domain pro-apoptotic protein) and VDAC1 (voltage-dependent anion-selective channel 1, an endogenous outer-mitochondrial membrane channel), resulting in the VacA-induced MOMP and subsequent release of cytochrome c [243]. Factors like the increased cytoplasmic pool of the apoptosis-inducing factor (AIF), seen after *H. pylori* exposure [256] or expression of cellular inhibitor of apoptosis protein (c-IAP)-2 upregulated during the early period of VacA stimulation [249], may also significantly influence the fate of the cell. Ultimately, *H. pylori* VacA causes the release of cytochrome c [189,210,238,243,255] and induces cell death.

## 5. Conclusions

The VacA protein is a very important virulence factor of the human pathogen, the *H. pylori* bacterium. It is a toxin secreted outside the bacterium that enters host cells and forms transmembrane pores in both the plasma membrane and organelle membranes. This review presents the route that VacA must follow from its synthesis in the bacterial cytoplasm to its incorporation into the membranes of host cell organelles. Reaching the target location requires overcoming several membrane barriers, which occurs using various mechanisms: classical export via the SEC translocon, T5SS secretion, penetration into the lipid bilayer thanks to regions capable of forming a membrane channel structure, endocytosis, and TOM complex translocation. The individual stages of this journey are accompanied by changes in the primary sequence of the VacA protein, which undergoes the sequential post-translational modifications, consisting in cutting off fragments of the polypeptide chain, which ultimately leads to the formation of the mature toxin. The release of the toxin into the extracellular environment enables it to adopt structures capable of interacting with the host cell membranes, and further conformational changes lead to the formation of transmembrane pores. Not all processes involved in the VacA transport have been understood. For example, the mechanism of pore formation in the membrane has not been characterized in detail to date. It is also unknown how exactly VacA enters the inner membrane of mitochondria. The proposed functions of VacA as a virulence factor are related to its ability to form transmembrane pores. These pores have the characteristics of chloride channels, which causes disturbances in the homeostasis of ion exchanges in the cell. Such channel activity leads to the formation of large cytoplasmic vacuoles and also causes disruptions in the functioning of endosomes, lysosomes, and mitochondria. In this way, VacA is thought to facilitate the successful infection of the gastric epithelium by *H. pylori*.

## Figures and Tables

**Table 1:** Presence of VacA in OMVs.

Strain	Ref.
CCUG 17874	[74] *
60190, 84–183	[75] *
4767-C, 2074-Cd	[76] *
NCTC11637	[77] *
CCUG 17875	[78] *
60190, SS1, SS1^s1i1^	[79] *
251 (and mutants: tolB, tolB+, pal, pal+, tolBpal, cagPAI)	[80]
HP99	[81] *
251 mutant cagPAI	[82]
B128_7.13	[83]
26695	[84,85,86]
NCTC11637, *H. pylori* 400 (CGMCC 15126)	[87]
TN2	[88]
60190	[89]

* Confirmed by specific antibodies.

## Data Availability

Data sharing is not applicable.
